# The physician shortage in Israel and a policy proposal for improvement

**DOI:** 10.1186/s13584-023-00552-1

**Published:** 2023-02-16

**Authors:** Yulia Treister-Goltzman, Roni Peleg

**Affiliations:** 1 Department of Family Medicine, Siaal Research Center for Family Practice and Primary Care, The Haim Doron Division of Community Health, Faculty of Health Sciences, Ben-Gurion University of the Negev, POB 653, 84105 Beer-Sheva, Israel; 2 Southern District, Clalit Health Services Community Division, Beer-Sheva, Israel

**Keywords:** Physician shortage, Policy proposals, Reform, Inequalities in health care, Professions in distress

## Abstract

**Background:**

There is a decrease in the supply of physicians in Israel resulting from the declining flow of immigrant physicians from the former Soviet Union, a large proportion of whom have reached retirement age in recent years. This problem could become worse because the number of medical students in Israel cannot increase quickly, especially because the number of clinical training sites is inadequate. The quick population growth and anticipated ageing will exacerbate the shortage. The aim of our study was to accurately assess the current situation and factors that affect it, and to propose systematic steps to improve the physician shortage.

**Main body:**

The number of physicians per capita is lower in Israel than in the OECD at 3.1 vs. 3.5 per 1000 population, respectively. About 10% of licensed physicians live outside of Israel. There is a sharp increase in the number of Israelis returning from medical school abroad, but some of those schools are of low academic standard. The main step is a gradual increase in the number of medical students in Israel with a transition of clinical practice to the community, and hospital clinical hours in the evening and in the summer. Students with high psychometric scores who were not admitted to an Israeli medical school would get support to study in quality medical schools abroad. Additional steps include encouraging physicians from abroad to come to Israel, especially in specializations in distress, recruitment of retired physicians, transferring functions to other medical professions, economic incentives for departments and teachers, and incentives to prevent physicians from quitting or migrating to other countries. It is also important to close the gap between the number of physicians working in central Israel and the periphery through grants, employment opportunities for physicians’ spouses, and preferential selection of students from the periphery for medical school.

**Conclusions:**

Manpower planning requires a broad, dynamic perspective and collaboration among governmental and non-governmental organizations.

## Background

The functional level of any healthcare system is dependent, primarily, on its human resources. One of the major threats is a shortage in professional manpower. Physicians are a dominant component of the healthcare system in terms of administration, budget, and treatment. In many OECD (Organization for Economic Co-operation and Development) countries the rate of increase in physicians per 1000 population has slowed down and a shortage in medical personnel is forecast. Thus, steps have been initiated in many countries to prevent a physician shortage and these steps have led to a slower decline or even a change in direction [1]. It is not easy to determine the appropriate number of medical personnel, especially doctors. The supply and demand is related to demographic trends (fertility, death, immigration), quality of life, public expectations and demands from the healthcare system, the structure of the healthcare system, patterns of utilization of medical services, and governmental technological, legislative, and budgetary processes. The balance between supply and demand for changes within countries is also associated with population distribution. Thus, the depth of personnel that is considered high in one country could be considered insufficient in another. The central driving force for increasing medical manpower in Israel was the waves of immigration, especially from the former Soviet Union in the second half of the 1990s. Since then, there has been a drop in the number of new licensees in Israel [1].

The aims of the present study were to assess the number of physicians in Israel, the factors that affect the balance between the number of physicians and the needs of the healthcare system and, based on these findings, to propose systemic solutions to address the physician shortage in Israel.

## Main text

### The number of physicians in Israel

**Fig. 1:**
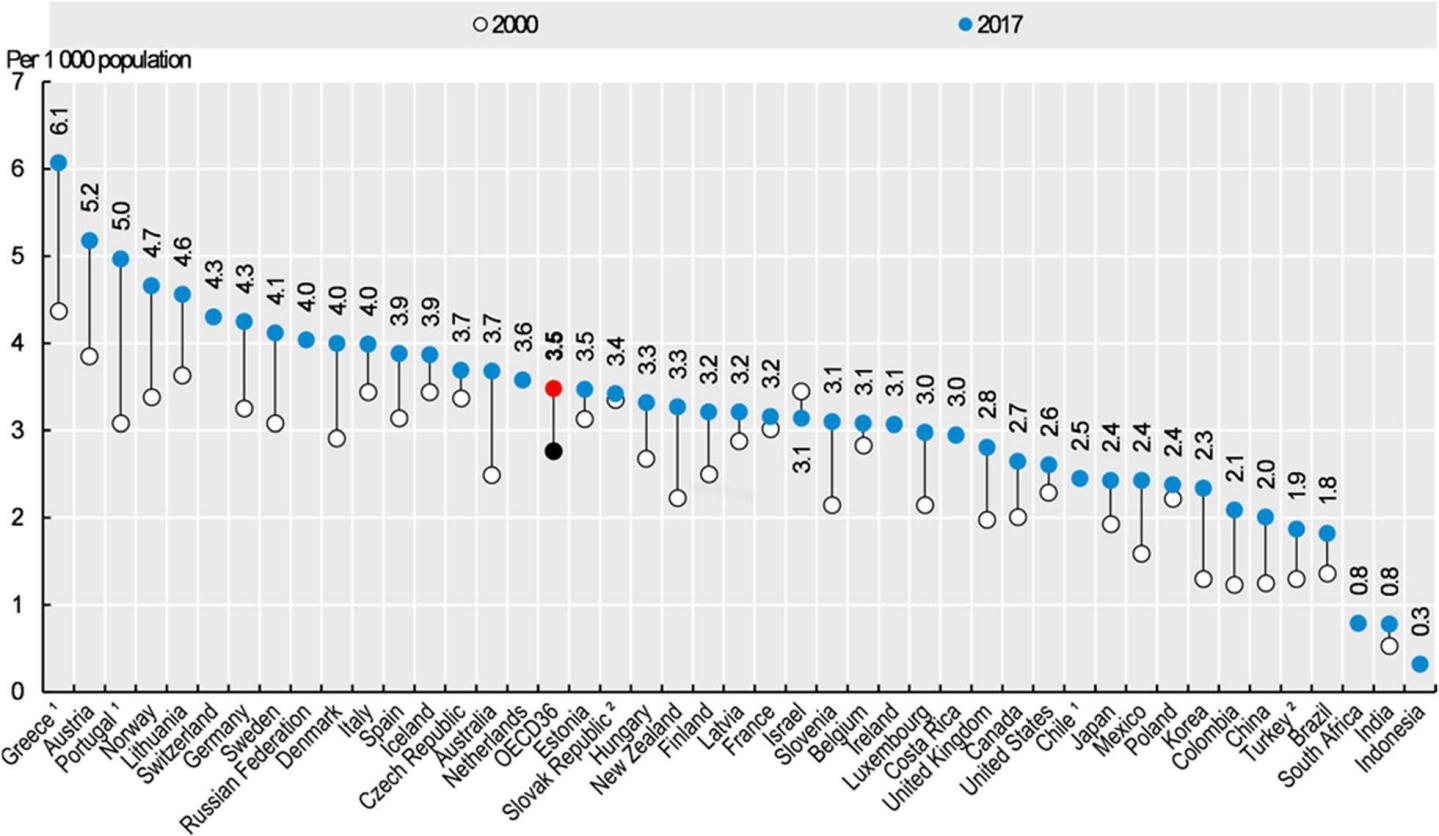
Practicing physicians per 1000 population, in the years 2000 and 2017 (from the OECD site)

**Fig. 2:**
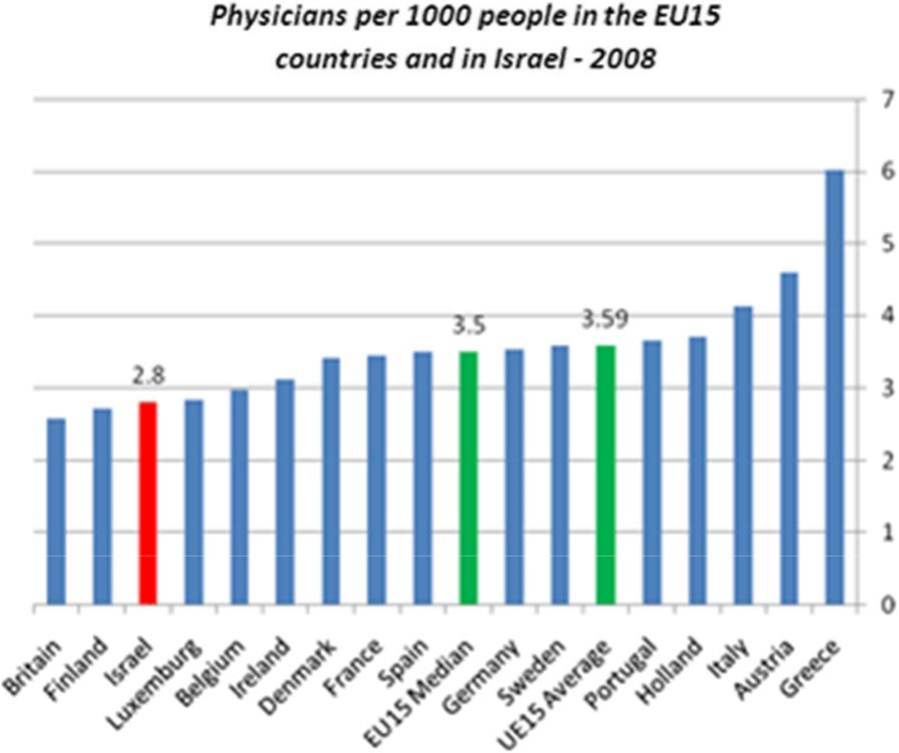
Practicing physicians per 1000 population in the 15 countries of European Union, in 2008

What is the ideal rate of physician in a country? A frequently used tool is comparison of the country’s rate with the mean rates in OECD countries. However, this tool is deficient and does not reflect the actual situation for several reasons:

1. The OECD includes countries that lag economically in comparison to Israel and countries that do not share Israel’s values and laws, such as the National Health Insurance law. One could get the wrong impression that the number of physicians per 1000 population is higher in Israel compared the OECD mean when the comparison is with all OECD countries [2] (Fig. 1), but when a group of investigators from the Israel Medical Association used this measure to compare Israel with 15 countries that are like Israel in terms of health and economics, Israel was ranked at the bottom of the list (Fig. 2) [3].
2. There is a difference in the definition of actively working physicians in each country. Israel, for example, defines working physicians as the mean number of physicians with an Israeli license below the age of 65. The resulting number is higher than the actual number of actively working physicians.
3. The measure does not consider differences in the allocation of manpower among medical specializations and geographical regions.
  To get the full picture, one needs to consider physicians weekly work hours, and the percentage of female physicians, many of whom tend to work in part-time positions with fewer weekly work hours [4]. The first step in any project is to assess the current situation. In Israel, in contrast to many other countries, a medical license is granted once in a lifetime without need for renewal. For many years the estimate of the number of physicians in Israel was based on Ministry of Health data on licensed physicians, which were updated in accordance with data on deaths from the Ministry of the Interior but did not relate to active practice. The Central Bureau of Statistics tried to reach a more precise estimate by issuing annuals reports on the labor force in the healthcare system, and the Ministry of Health has improved its estimate starting in 2010 by collecting data from Israeli Health Maintenance Organizations (HMO) and hospitals. This information was partial and based on small samples. The results of a more comprehensive study were reported in 2017 [4]. The results of this study emphasized additional weak points besides the physician shortage. Only 74% of licensed physicians (about 24,000) live and work in Israel. The percentage of female physicians is about 41%, which is higher than the rate in the United States where only a third of the physicians are women. Most Israeli employers define a full-time position as 42–45 h per week. Close to 30% of the physicians in Israel work fewer hours. In all age groups the percentage of female physicians who work less than full time is higher than for male physicians. Another finding of concern is that 10% of licensed Israeli physicians live and work abroad, and the numbers are higher for specializations in distress such as anesthesiology (15%) and pediatric psychiatry (12%) [4]. Another unique feature is that a large proportion of Israeli physicians graduated from medicine schools outside of Israel. In 2017, 59% of the recipients of a medical license received their medical degree abroad [5].

Thus, the low absolute number of physicians per capita in Israel (3.3 per 1000 population in 2019) relative to other developed countries and the OECD average (3.5 per 1000 population) [2], the features of the Israeli physicians' workforce described above, the downward trend and estimation shortcomings, turn the number of physicians to not sufficient.

The demand for and use of healthcare services in general and the number of physicians in general may increase in coming years for several reasons. First, the population growth is predicted by the Central Bureau of Statistics to reach 13.5–16.8 million residents by 2035 [6]. Although Israel is a relatively young country, its life expectancy rate is high and its medical level is advanced, so the elderly population is expected to increase by 77% between 2015–2035, and the rate of increase among the elderly is expected to be 2.2 times higher than the general population [6]. This change in the composition of the population will lead to an increase in the burden of chronic diseases. The rate of hospitalizations among the elderly is 50% higher than the rest of the population, and the rate of appointments with family physicians and specialists is 6–8% higher [6]. Thus, the increased size of the general population and among the elderly will exacerbate the physician shortage and cause a greater burden on the medical staff overall, and on physicians in particular.

### Recommendations of committees that have assessed the physician shortage issue

Awareness of the worsening physician shortage has led to the commissioning of several committees over the last two decades [5–7].

1. The Pazi committee, which delivered its findings in 2002, recommended increasing the number of medical students to 900 each year, representing an additional 600 students.
2. The Halevy committee, whose recommendations were submitted to the Planning and Budgeting Committee of the Council for Higher Education in 2007, recommended increasing the number of medical school graduates each year and the establishment of a new medical school.
3. The committee appointed to assess the future manpower needs of the healthcare system, headed by Gabi Ben Nun, which submitted its report in 2007, recommended increasing the number of institutes for physician training.
4. A 2010 committee for manpower planning also recommended increasing the number of medical students in Israel to 700 per year, incentivizing physicians to train in specializations in distress, reducing physician emigration through incentives, and establishing a headquarters for ongoing evaluation and planning of medical manpower in the Ministry of Health. This committee concluded that without intervention the number of physicians per 1000 population would decrease in Israel to 2.6 by 2025 [6].

Implementation of the recommendations of these committees has, indeed, led to a gradual increase in the number of medical students and the establishment of new medical schools in Safed in 2011 [7] and in Ariel in 2019 [5]. In 2020 the number of medical students increased to 825 per year [8]. A four-year medical degree program that trains 60 students each year has been functioning in the medical faculty of Tel-Aviv University since the academic year 2008–2009. It is open to students with a bachelor’s degree in the natural or life sciences and exact sciences or engineering, who completed all core subjects as a condition for application. Since 2009–2020 the Hebrew University in Jerusalem has run a 6-year program to train 50 medical students for service in the army as part of the army’s future cadre of physicians [1].

### Existing programs to encourage immigration of physicians to Israel

A special mention should be made of the “Masa” project. This very successful project, which has been active from 2009 is comprised of several training programs lasting from one semester to one year, for young Jewish individuals (18–30 years) who live outside of Israel [9]. This program provides an example of successful collaboration among the Jewish Agency for Israel, the Ministry of Health, and the Ministry of Finance. It includes the study of various subjects, including intensive Hebrew studies, excursions, experiences, and learning about Israel. This enables smoother integration into Israel and the recognition of foreign diplomas prior to immigration to Israel. Participants in the program receive grants and scholarships based on their country of origin and their economic state. Much of the program is directed at graduates of medical school. About 80 medical school graduates take part in the program annually and 85–98% immigrate to Israel at some point. A quarter of these physicians work in the periphery in the north and south of Israel and many work in specializations in distress. More than a third joined the Israeli army prior to the start of their specialization training and remained in the medical corps [10]. This is a unique program with double value, as it addresses the needs of the healthcare system and serves as a Zionist project that brings together the best Jewish youth in the Diaspora and the State of Israel.

The Jewish Souls United program recruits new immigrants among North American Jews and facilitates their immigration process [11]. It has helped medical personnel including physicians to immigrate to Israel by expediting the procedure for recognition of diplomas so that the physicians can be integrated into the system within months of arrival in Israel. In the wake of the waves Covid-19 pandemic around the world, which caused the death of thousands of Jews in the United States and highlighted the failings of the US healthcare system, about 200 medical personnel immigrated to Israel including 61 physicians, 29 nurses and 108 from paramedical professions [12]. However, there are no exact data on the number of physicians and other medical personnel from the Jewish Souls United program who remained in Israel.

### Graduates of medical schools from abroad, the Yatziv reform and its anticipated effect

In recent years unexpected changes have led to an increased number of physicians in Israel. The number of Israeli graduates (Israeli-born or immigrants) from foreign medical schools was 763 in 2018 compared to 195 in 2010 and 111 in 2005. As a result, the number of physicians below 67 increased to 3.3/1000 population [6], but this trend does not seem sustainable. In 2019, following many reports on a low professional level and inadequate training of graduates of some foreign medical schools especially from developing countries, Prof. Yatziv who heads the Ministry of Health’s licensing bureau, began to develop a comprehensive reform to assure the professional level of physicians receiving licenses outside of Israel. Even if these graduates pass the licensing test in Israel, the percentage that pass specialization tests is less than that of graduates of Israeli medical schools [5]. The effect of this reform will be incremental and will reach its peak in 2026, leading to a decreased rate of growth in the number of physicians in Israel [8].

In March 2021, the director of the Financial & Strategic Planning Administration of the Ministry of Health published a forecast for the number of physicians/1000 population by 2035, which was based on growth trends of the previous years and factored in the anticipated effect of the reform program to assure professional competence. According to this forecast the number of physicians in 2025 will be 3.3/1000 and will decrease to 3.22/1000 in 2035, primarily due to the anticipated decrease in the influx of students from abroad. After factoring in the ageing of the population, the adjusted number of physicians is predicted to be 3.03/1000 in 2035, which is significantly lower than the number today and the mean number in the OECD [8, 13].

### Recommendations for ways to improve the situation

**Fig. 3:**
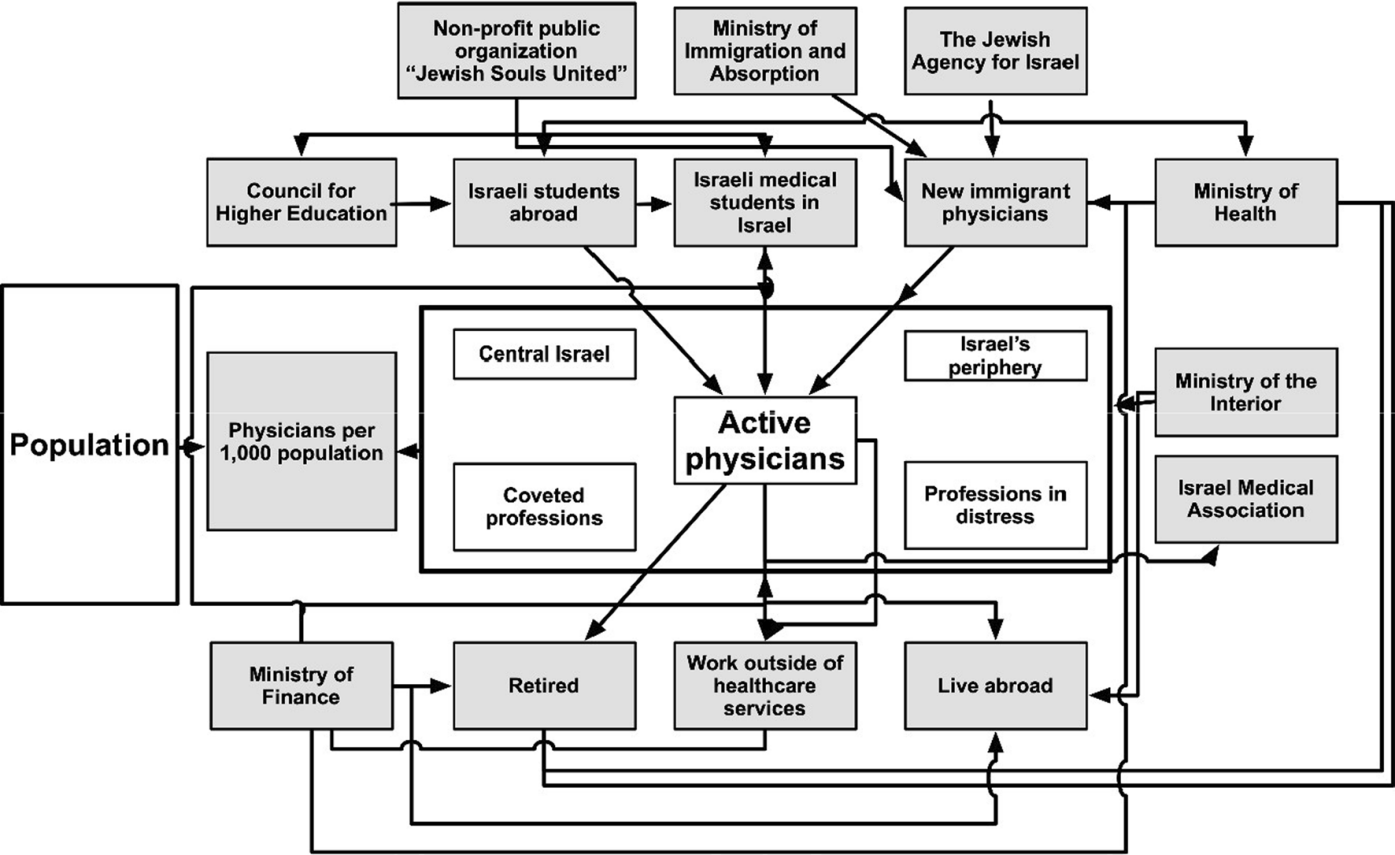
A conceptual model depicting the multitude of factors associated with the number of physicians

Figure 3 shows a conceptual model depicts the main factors that affect the number of physicians in Israel and the involved organizations. By influencing each of the six arms associated with the number of active physicians in Israel it will be possible to improve the rate of physicians per population. Thus, this model can serve as the basis for the development of a systematic intervention plan to increase the rate of physicians, their unique sociodemographic makeup, and the mix of active physicians as described above.

#### Increasing the number of medical students in Israel

Considering the high professional level of graduates of Israeli medical schools, increasing their number is the preferred way to increase the number of physicians per 1000 population. Despite the widespread perception that the training of medical students in Israel is very costly, it is at the same level as natural science degrees and lower than the cost of training physicists [5]. The main obstacle to training medical students is the need for clinical training settings in the last three years of study and the year of internship. This training usually takes place in hospitals, a setting that is overburdened with routine work even without the additional burden of teaching. Compared to hospital physicians, community physicians see the full picture of morbidity and a broader range of clinical conditions. Shifting the paradigm and planning the transition of some hospitalized patients to community settings such as home care can be effective, prevent hospital-acquired infections, are cheaper for the health care system, and make the patient and family feel more comfortable and at ease [6]. On the other hand, this type of transition is personnel intensive and requires mobile resources. Home care teams must be able to immediately access and deliver all needed care, including diagnostics, monitoring, pharmaceuticals, and nursing services. It also requires physicians who are adept at working with home-based patients while coordinating all aspects of care. Patient intake and discharge must be handled promptly, including care plans for the patient during home hospitalization and transitioning patients to their regular providers after the acute phase. Other steps that could be taken include planning to use unutilized hours for clinical practice such as afternoon hours and the summer. The Glaser-Israeli report in 2012 [14] showed that although funding for clinical clerkships was transferred from the Office of Higher Education to hospitals, there was no direct compensation, or addition of manpower to the teaching departments, so they were not able to shoulder the burden of teaching students. Monitoring this process and the direct compensation for extra hours of teaching in the evenings, summer, etc. could improve teaching capacity in those departments. Despite the physician shortage, the training period for physicians in Israel is the longest in the west, continuing for seven years in contrast to 4.5–6 years in other western countries. Based on the reform proposed by Prof. Zion Hagay, the head of the Israel Medical Association, the length of medical studies would be shortened by incorporating 6 months of the internship year, which consists of 11 months of practical experience in different wards, into the 6th year of medical school and the other six months of internship would be recognized as part of residency training, as is practiced in the United States [15]. Reducing the length of medical school in this way led to a significant increase the number of physicians per 1000 population in Germany from 3.1 in 2000 to 4.1 in 2017 [15]. Graduates of foreign medical schools would still be required to complete six months of internship in Israel for a basic practice experience. We support this program considering its success in other countries, and its contribution to increasing the rate of absorption of medical graduates in Israel into the system.

1. *Recommendations for guidance, monitoring, and improved absorption of students studying in foreign medical schools*. Because of the high threshold for admission in medical school in Israel and the inability achieve a significant increase in the number of students who study in Israel, some Israeli students will continue to study abroad. In wake of the Yatziv reform the first steps have been taken to monitor the situation, and diplomas from three foreign institutes have already been disqualified. The World Federation for Medical Education [16] is an international organization that is conducting a process of accreditation of medical schools throughout the world. Its goal is to establish an international standard for medical studies [16]. Beginning in 2023, the United States, for example, will not allow graduates of institutes not recognized by the WFME to take licensing exams. Israel can adopt this approach. An alternative would be to continue to assess medical institutes abroad by considering the percentage of candidates who passed licensing examinations in previous years. Among medical students studying abroad there is a fraction of Israeli students with high psychometric scores (over 700) who did not meet the admission requirements of Israeli medical schools. These students could be provided with grants or scholarships to study in good medical schools abroad. This strategy enabled countries such as Norway, Sweden, and Singapore to significantly increase the number of physicians [17, 18]. To prevent physician drain after medical school these students could be required to commit to a specified number of years of work in Israel.
2. *Encouragement of immigration of physicians to Israel*. Collaborative projects among the Ministry of Health, The Ministry of Immigration and Absorption, the Jewish Agency for Israel, and voluntary organization such as Jewish Souls United were described above. Since these projects have proven successful, additional projects should be developed to identify and encourage Jewish physicians in the Diaspora to immigrate to Israel, especially those who work in specializations in distress in Israel. This can be accomplished through financial incentives, reduction of the bureaucracy involved in getting Israeli certification, help in finding work in Israel, and help in housing and absorption into Israel for the physicians’ families.
3. *Activation of retired physicians*. The retirement age to qualify for a pension in Israel is 67 years for male physicians and 64 years for female physicians. In 2018 the Civil Service Commission reached a decision to enable governmental hospital to recruit retired physicians in an attempt to alleviate the physician shortage, especially in professions in distress [19]. Retired physicians who specialize in professions in distress are employed in part-time positions up to half time till age 70, maintaining the last rank they held prior to retirement. Special approval is also available to employ these physicians for more than a half-time position and for a period beyond three years [19]. For independent physicians the protocols of the HMOs are more flexible and enable continued work for various time periods after retirement age, from eight years and even more for different HMOs. The is no regulation that limits the employment of independent physicians in the community.
  This policy can be carried a step further. Life expectancy and general health are continually improving in Israel. Many retired physicians are still in a state of good physical and mental health and can continue to work. Their knowledge and experience are a valuable asset not only to reduce the burden of work in the hospital or community, but they can contribute to the education and training of future generations of physicians.
  The possibility of employing retired physicians is very relevant for Israel considering the relatively large proportion of physicians in Israel who are over the age of 45 (69%) compared to the OECD (56%) [5]. This situation has developed because of the large wave of immigrant physicians in the 1990s who are now approaching retirement age. It is forecasted that 800 physicians will reach retirement age annually for the coming years. This accelerated retirement rate is expected to continue for another five years and then decline gradually [8]. The employment of retired physicians in HMO hospital and community clinics, and extension of the employment age can help alleviate the physician shortage.
4. *Preventing physicians from quitting the field of medicine or immigrating to other countries*. The reasons that lead physicians to quit the profession or to immigrate to other countries are no different from other places in the world and include burnout, difficult work conditions, and erosion of physicians’ autonomy. In most cases the reasons for emigrating are not due to a negative attitude to the profession, but to poor working conditions [2]. Israeli physicians report a low level of satisfaction with physical conditions at work, their wages, and the lack of balance between personal life and work [20]. Many physicians point to the lack of opportunity for professional development in Israel and the lack of attractive positions for sub-specialization [20].
  About 10% of Israeli physicians of working age live abroad [4]. As in a vicious cycle, one of the reasons for difficult work conditions is the physician shortage. A campaign to reduce long work hours and the number of monthly nights on call has been led by the “Mirsham” organization that numbers about 1000 residents [21]. Improvement in work conditions could prevent physicians from quitting the profession or immigrating to other countries. Although shortening shifts from 24 to 16 h could have a positive effect on the balance between residents’ personal lives and work, it would have significant financial ramifications for hospitals. It necessitates an increase in manpower that might not be available, at least in the short-term, especially in smaller hospitals in the periphery.
5. *Increasing the number of physicians working in the periphery and in professions in distress to close the inequality gap*. The Ministry of Finance, the Ministry of Health, and the Israeli Medical Association started a medical grant program for physicians in 2011 as part of a broader project to encourage physicians to work in the periphery [22]. The project, although applied only intermittently, succeeded in recruiting physicians, particularly for specializations in distress, to the periphery. Thus, it makes sense that other similar projects with incentives to specialize in professions in distress and to work the in periphery be implemented. Additional steps would be to actively aid physicians’ spouses in finding work and housing. There is a greater probability that physicians who are residents of the periphery will work there following graduation from medical school. Thus, providing aid for residents of the periphery for medical studies could help increase the number of physicians working in the periphery.

### Prof. Gamzu’s committee and initial steps already implemented

In 2022 the recommendations of an inter-institutional committee initiated by the National Institute for Health Policy Research and headed by Prof. Roni Gamzu, whose mandate was long term planning of medical manpower in Israel up to the year 2035, were published [23]. After a review of the current manpower situation in the healthcare system using precise numbers, concrete steps were proposed to reduce the physician shortage. The annual number of recipients of licenses to practice medicine in 2035 would be 2,000; 60% graduates of Israeli medical schools. The number of Israeli medical school graduates is already increasing. An increase of 80 students per year is expected over the coming years. A gradual reduction of programs for foreign students that can enhance the process is already underway. As a result of this reduction, from October 2023 there will be an additional 130 Israeli medical students. Particular attention has been paid to the regulation of academic positions and the development of an appropriate infrastructure to increase study groups, expand the number of clinical study settings by teaching in the afternoon hours, and teaching in the community setting. A uniform remuneration model will be built to incentivize hospitals, hospital departments, and instructors to increase the number of students by allocating resources at the level of departments and doctors involved in teaching, and not just providing the resources to hospital administrations [23]. The number of teaching weeks in the primary hospital departments (pediatrics, gynecology, surgery, and internal medicine) will be increased to 35 weeks per each year and there will a minimum number of eight students in each group in clinical settings in the departments [23]. Given that there are 52 weeks in a year, there may be a need to increase the number of senior physicians in each department and provide them with academic appointments so that these valuable teaching resources can be utilized to the maximum.

The committee’s program relates to the gap in medical manpower between central Israel and the peripheral regions. The main intervention proposed by the committee to reduce these gaps is reduction in the infrastructure gap, in particular hospital beds. Every planned addition of beds, especially hospital beds, will focus on the Negev and the Galilee regions since allocation of doctors to different regions depends on the supply of beds in those regions. The Ilanot program was launched in 2022 to increase the number of students who live in regions with physician shortages [24]. Sixty medical students, 30 each from the north and from the south, either born in those regions or with a strong connection to them, are beginning medical studies with a full scholarship covering tuition and living expenses, and extra-academic precepting including training in medical management, etc. The Ilanot program is expected to expand gradually to 100 students, 50 each from the Negev and the Galilee regions by 2025. There is also a plan to increase the number of doctors from the ultraorthodox Jewish community. In 2022, in collaboration with the Kemach Foundation, a program was started to reduce the unequal representation of the ultraorthodox sector in the medical field [23, 25]. Twenty students began a four-year program, with an emphasis on female students with an appropriate first academic degree. For medical specializations, where there is a manpower gap stemming from a lack of demand for specialization in the profession, the Ministry of Health was mandated to plan a registry of such specializations which are deserving of and suitable for special support. The Ministry set up a repository of information on residents at any given time. Based on a deep analysis of the needs of the healthcare system in Israel in terms of the number of specialists in each field, and in close collaboration with hospitals and HMOs, annual national goals will be set by the Ministry of Health for the number of new trainees in each field of specialization. The Scientific Council of the Israel Medical Association will be responsible for execution of the program [23].

Another step that was emphasized by the Gamzu Committee and that could reduce the physician shortage is the transfer of authority in some areas to allied medical profession other than doctors, especially nurses. The role of clinical nurse specialists emerged in the United States in the twentieth century and was implemented successfully in European and Asian countries in the 2000s [26]. A unique track was developed in Israel that enables senior nurses with a second academic degree or more and appropriate training, to specialize in specific nursing fields. This role was recognized in the Public Health regulations (confirmation of a specialist degree in nursing) including the training process and licensing of nurse specialists and definition of the approved fields of specializations in Israel [27]. The main functions of specialist nurses includes: examination of the patient; assessment of the patient’s condition; referrals for diagnostic testing and follow-up and for medical and paramedical consultations; formulation of treatment plans and treatment orders; management of drug therapy; symptom control; conduct of medical procedures within the bounds of delegated authority; referral of patients with ongoing care and medical work-up; guidance and counseling for patients and families; and telephone or online consultation and treatment. Specialist nurses can function in a spectrum of settings including the hospital, community clinics, the patient’s home, and telephone and online consultations and treatment. Some of the specializations were already approved in 2013 (supportive care, geriatrics, and diabetes mellitus) and some (wounds, psychiatry) are in stages of development [23].

The recommendations of the inter-institutional committee were aimed at slowing down the rate of decrease in the number of physicians that resulted from the Yatziv reform. Without these multifaceted interventions, the projected number of physicians in 2025 would have been 3.3/1000 and would have decreased to 3.03/1000 in 2035 [8, 13]. Implementation of the recommendations of Roni Gamzu’s committee would raise the absolute number of physicians to 3.4 per 1000 population in 2035, while maintaining the quality of the physicians and providing a better allocation of manpower among medical specializations and geographical regions [23]. Still, the number would be slightly lower than the OECD average.

### Proposals for new medical faculties

Two new initiatives have been put forth as a part of the overall movement to increase the number of medical students in Israel. The first is the initiative to establish the first private medical school in Reichman University, which is already in advanced stages of development, but has not yet received legislative approval [28]. The establishment of this school became possible through a donation from the Recanati family and it has signed cooperation agreements with the Sheba and Beilinson medical centers. The second initiative is to open a new medical school with a unique "boutique" program of physician-researchers (MD-PhD) in collaboration with the Sheba Medical Center [29]. In the first phase, the program would include about 40 students per year, and would be a six-year program intended for students with bachelor or advanced degrees. Research activity would be carried out by students at the Weizmann Institute, and clinical training would be carried out at the Sheba Medical Center. This initiative is intended to create a direct connection between science, in particular basic research, and the world of medicine, and to train research doctors who would enrich hospitals with holistically trained personnel bringing the most advanced and innovative treatments to patients on the one hand, and enriching scientific research carried out at the Weizmann Institute, on the other. While this initiative could possibly exacerbate the deficit in the clinical infrastructure, which is traditionally considered a bottleneck, the authors believe that the extension of clinical studies to the afternoon hours and cancellation of international programs for medical studies could make this initiative feasible.

## Discussion

This paper focuses on the complexity of the issue of the number of physicians in Israel, which is comprised of the existing mix of physicians and the balance of physician immigration and new graduates of medical school with physicians retiring, quitting the profession or emigrating from Israel. The conceptual model (Fig. 3) depicts the multitude of factors associated with the number of physicians and the large number of involved governmental and non-governmental organizations, which play an influential role in each of its arms. The emphasis is on a gradual increase in the number of students in Israel with an expansion of clinical settings for students beginning with practice in the community in the fourth year, transfer of some study hours to the evening and the summer, and support for students with a high psychometric score who were not admitted to medical school in Israel so that they can study in the best schools abroad. Additional steps to improve the situation include monitoring and quality assurance for students who studied abroad and are entering the Israeli healthcare system, encouragement of physician immigration from around the world, especially for specializations in distress, economic incentives for departments and teachers, recruitment of retired physicians, prevention of physicians quitting the profession and of physicians emigrating to foreign countries, and reduction of the gap between central Israel and its periphery.

It is important to realize that there is a gap of 15–20 years between the implementation of policy steps today and a change in the number of physicians per 1000 population, which is affected only slowly by the change in the ratio of physicians leaving and entering the healthcare system. This change is affected by many factors, of which a critical one is the time required to complete medical studies, with the addition of 5–9 years for specialization training. Increasing the number of physicians entering the system will necessitate the establishment of additional positions for physicians, residents, and specialists, through collaboration with the Ministry of Health and the Ministry of Finance, which plays a dominant role in the entire process of policy change. Physicians, who are the most expensive resource in the healthcare system, are often required to take part in activities that do not reflect their professional skills, for example drawing blood from patients in the hospital, and dispensing prescriptions for chronic medications in the community. These activities can be carried out by others in the healthcare sector, particularly nurses and physician assistants (PA). The nurse shortage in Israel is even more severe than the physician shortage, so the transition of nurses to these tasks could exacerbate the nurse shortage. The Gamzu committee solidified steps for the development of clinical nurse specialists, but there are other allied professions including pharmacists and physician assistants that, after additional training, could take on medical functions that are only carried out by doctors today. The development of the PA profession is inadequate. Experience in other countries indicates that PAs can improve access to care, reduce errors, increase efficiency, and play significant roles in healthcare systems. In Israel, to date, PAs have been added to emergency departments by training paramedics for this function. They seem satisfied with this new role, reporting personal fulfillment, improved career prospects, and wage improvement as leading reasons for job or role satisfaction [30]. PAs and their counterparts offer flexibility in adaptation and deployment in diverse settings. This is facilitated by the educational system, since adjusting a PA program’s didactic and clinical syllabi is easier than changing the medical school curriculum. The physician’s face time with patients is already too short for effective patient-centered care. Clinical scribes can assist in gathering and documenting information, allowing physicians to stay away from the computer screen and focus on the patient’s narrative. On the other hand, the intimacy, even the sanctity, of the few minutes patients spend in private with their physician could be disrupted [31]. The Israeli health system still needs to determine if adding to the role of PAs and introducing scribes will be accepted by the public and by physicians. Employing more technicians and more secretaries could also maximize physician productivity and decrease the non-professional workload. Thus, further systemic change is needed.

### Study limitations

In this review we have discussed a complex situation that involves many governmental and non-governmental organizations and is affected by a multitude of factors. There are many other variables that affect the Israeli workforce that are beyond the scope of this article. The fiscal ramifications of currently implemented and future multifactorial broad, dynamic changes are also beyond the scope of this article.

## Conclusion

The physician shortage is a multifactorial problem that requires a broad perspective, recognition of the unique situation in Israel, planning and updating of dynamic steps. It is influenced by the situation in other Israeli healthcare sectors and requires collaboration among multiple organizations, taking into account the growth and dispersion of the population.

## Data Availability

Not applicable.

## Abbreviations

OECD: Organization for Economic Co-operation and Development
HMO: Health Maintenance Organizations
PA: Physician assistant
